# Concentration Dependence of TiO_2_ Nanoparticles in Carbon Xerogels on Adsorption–Photodegradation Applications

**DOI:** 10.3390/gels9060468

**Published:** 2023-06-07

**Authors:** Anam Safri, Ashleigh Jane Fletcher

**Affiliations:** Department of Chemical and Process Engineering, University of Strathclyde, Glasgow G1 1XJ, UK; anam.safri@strath.ac.uk

**Keywords:** carbon xerogels, photocatalyst titania, adsorption, photodegradation, adsorption isotherm, recyclable composites, water treatment

## Abstract

A suite of composite materials comprising carbon xerogel content and TiO_2_ was synthesised via a modified sol–gel method. The textural, morphological, and optical properties of the composites were extensively characterised and correlated with the observed adsorption and photodegradation performances. The homogeneity and porous structure of the composites depended on the amount of TiO_2_ deposited in the carbon xerogel. During polymerisation, Ti-O-C linkages were formed, which favoured the adsorption and photocatalytic degradation of the target methylene blue dye. Adsorption was deemed favourable, and most accurately fitted by the Sips model, exhibiting a maximum uptake of 209 mg g^−1^ estimated for the sample containing 50% TiO_2_. However, the synergistic effect of adsorption and photocatalytic degradation for each composite depended on the amount of TiO_2_ deposited in the carbon xerogel. The dye degradation process for the composites with 50%, 70%, and 90% TiO_2_ improved by 37%, 11%, and 2%, respectively, after exposure to visible light after adsorption. Repeated runs demonstrated over 80% of activity was retained after four cycles. Thus, this paper provides insight into the optimal amount of TiO_2_ required within such composites for maximum removal efficiency via adsorption and visible light photocatalysis.

## 1. Introduction

Increasing water pollution, along with the appearance of emerging pollutants, has led to ongoing developments in innovative wastewater treatment methods that can meet the standards for clean water. Environmental catalysis is one such technology that can effectively respond to this demand, and these methods can be enhanced by developing new materials and processes to meet the needs of an increasingly industrialised society. Among these technologies, photocatalytic processes are interesting systems, which are occasionally used in combination with other techniques to improve water treatment processes. In particular, since these systems use visible irradiation, they can save energy, allowing them to be employed in developing countries. In this regard, the photocatalyst TiO_2_ has been reported in combination with other materials to enhance its photocatalytic performance. One way to improve photoactivity is by combining TiO_2_ with an adsorbent material, whereby the synergistic effect of the integrated materials enhances pollutant adsorption and disintegrates pollutants under visible light [1].

Carbon-based adsorbents are often used as adsorbent materials because of their high surface area and porous nature, which facilitate the adsorption of pollutants [2]. Additionally, carbon materials have been employed to modify the electronic structure of TiO_2_ to improve visible-light photocatalysis because TiO_2_ has a large bandgap and is only activated upon UV irradiation to generate electron and hole pairs, which undergo a series of chemical reactions to produce hydroxyl species responsible for disintegrating pollutants [3]. Additionally, carbon materials can entrap photoexcited electron and hole pairs, inhibiting their recombination and promoting charge transfer efficiency. Carbon gels, derived from the polycondensation of resorcinol and formaldehyde, have been investigated extensively for a range of applications in various sectors because of their tunability, large surface area, interconnected porous network, and high electrical conductivity [4]. Substantial research has been conducted on energy storage applications [5], gas storage [6], and thermal conductivity. Owing to their three-dimensional structure, which can serve as an ideal host for catalytic species, carbon gels have recently been used in combination with other materials in water treatment applications [7]. For water remediation applications, titania/carbon aerogel composites have been reported to successfully degrade dyes. These studies established that the synergy of mesoporous carbon and titania results in enhanced dye degradation when compared with other carbon/titania composites [8,9,10].

In our previous studies, we established that the high surface area and porosity provided by the carbon xerogel (CX) matrix enhanced the adsorption capacity of pollutants and modified the band gap of TiO_2_ due to chemical linkages formed between CX and TiO_2_, which promoted the photogenerated charge recombination rate for sufficient production of hydroxyl species. Therefore, the synergistic effect of combining these phases resulted in enhanced adsorption and photodegradation under visible-light irradiation. Overall, the composite synthesised with 10% TiO_2_ showed 72% degradation activity, which improved with further addition of TiO_2_, exhibiting 99% degradation activity for the composite with 30% TiO_2_ [11,12]. In another study by Garcia et al. [13], the successful synthesis of carbon xerogels and TiO_2_ composites showed significant degradation of the orange G dye. Studies so far have reported up to 40% TiO_2_ in the gel matrix, showing an increase in removal efficiency with increasing TiO_2_ content. To the best of our knowledge, no literature has been found on the application of samples with higher loading of TiO_2_ in the CX matrix, synthesised by the approach employed in this work. Hence, in this work, we synthesised a suite of CX and TiO_2_ composites (CXTiX, where X denotes % TiO_2_) with 20% sequentially increasing steps, starting from 50% TiO_2_. The composites were tested for their adsorption–photodegradation performance for the reduction in methylene blue (MB) dye. The dye degradation performance was analysed based on the structural, textural, and optical characteristics of the synthesised CXTi composites.

## 2. Results and Discussion

### 2.1. Characteristics of CXTi

The amount of TiO_2_ present in each composite was determined via thermal gravimetric analysis (TGA) after combustion of the organic phase in air. The recorded residual masses of the samples were slightly higher than the theoretical TiO_2_ contents, which can be ascribed to contributions from the segments of the RF phase trapped in the TiO_2_ phase. In contrast, TiO_2_ samples with very high amounts of TiO_2_, for example RFTi90, showed a residual inorganic phase, slightly lower than the theoretical amount, which may result from insufficient hydrolysis and condensation of the TiO_2_ precursor during material synthesis. Nevertheless, the experimental data are close to the expected values (Table 1). The arguments supporting the observed differences are in agreement with previous work for composite systems with Ti/carbon and Ti/epoxy resins [14,15,16].

**Figure 1:**
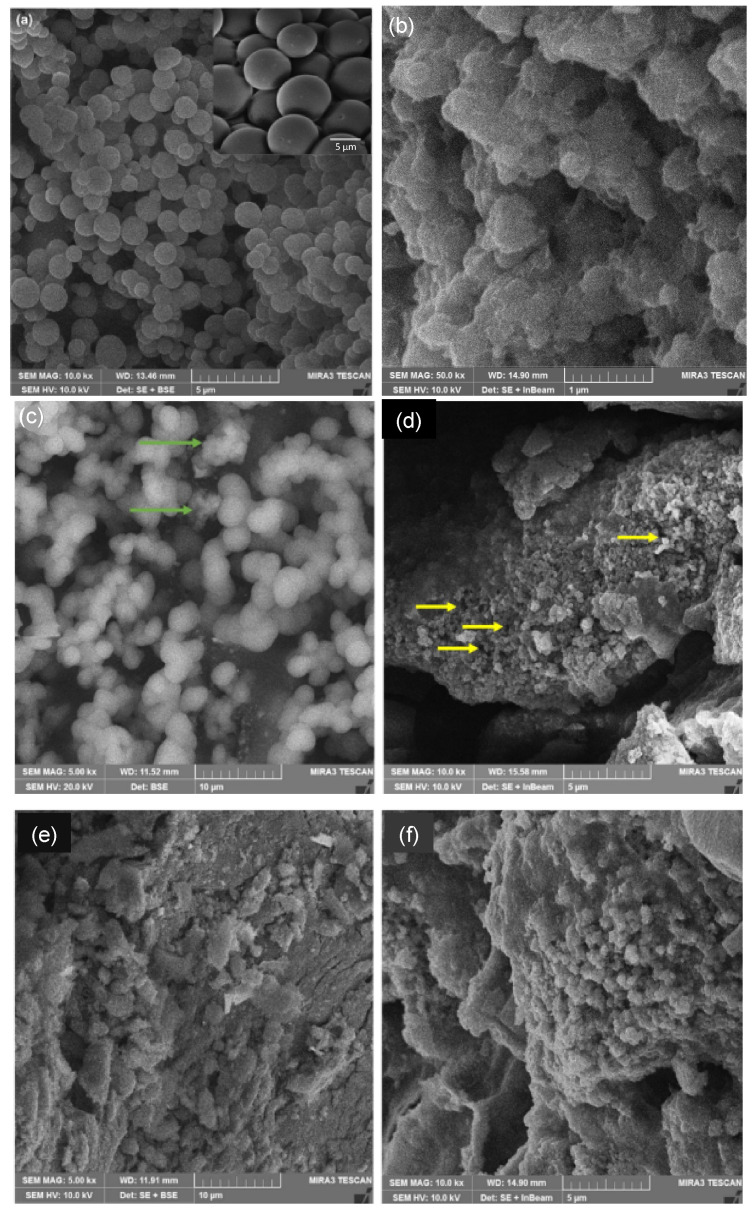
FESEM micrographs of (**a**) CX; (**b**) CXTi50; (**c**) and (**d**) CXTi70; (**e**) and (**f**) CXTi 90. Green arrows identify areas of greater surface roughness, and yellow arrows highlight reduced porosity.

**Figure A1:**
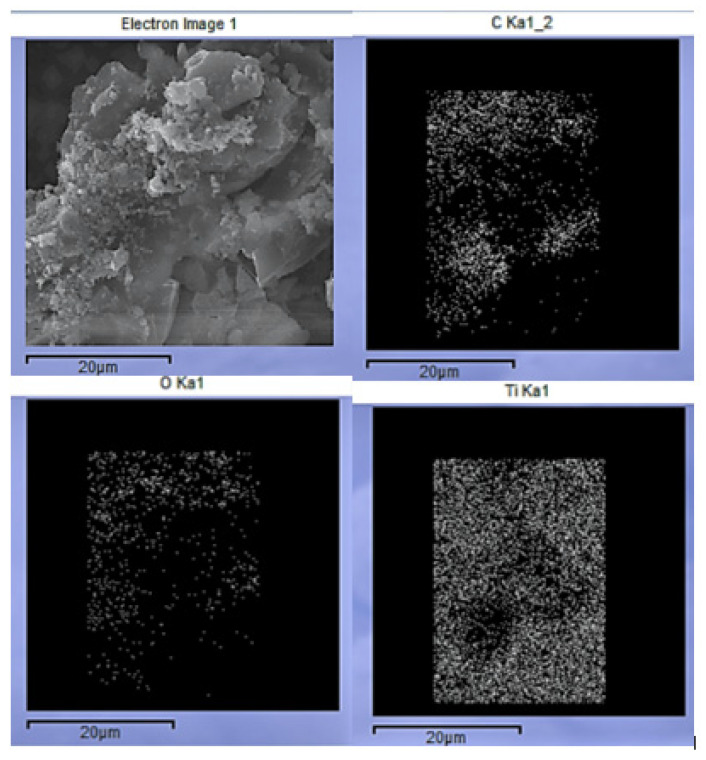
Elemental mapping of CXTi90.

In our previous work, for samples with low TiO_2_ content (10 and 30% TiO_2_ in CX), the composite samples maintained a regular spherical shape with an overall homogeneous smooth surface without differentiation between the organic and inorganic phases within the composites [11,12]. In this study, heterogeneity and surface roughness were observed, as smooth CX spheres (Figure 1a) seemed to be shielded with TiO_2_, seen in the micrograph obtained for CXTi50 (Figure 1b). With further addition of TiO_2_, the heterogeneity increased owing to reduction in the organic phase, as well as the tendency of TiO_2_ to aggregate, resulting in a heterogeneous distribution of TiO_2_ clusters. The TiO_2_ crystallites continued to grow, as shown in the micrographs for RFTi70, demonstrating an increase in TiO_2_ aggregates with greater surface roughness (green arrows) and reduced porosity (yellow arrows), in comparison with the highly porous, smooth carbon surface of CX and CXTi gels with low amounts of TiO_2_. Elemental mapping is included in Figure A1 (Appendix A). In the case of CXTi90, the discreteness of the carbon spheres became less evident, owing to the high TiO_2_ content, and the pores could not be identified through the micrograph images obtained for this sample (Figure 1e,f).

**Figure 2:**
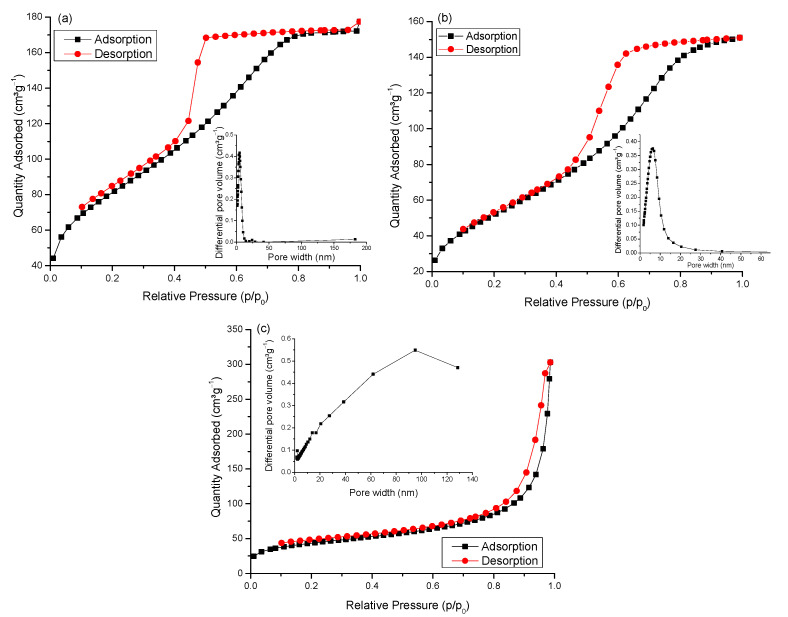
N_2_ sorption isotherms (77 K) and BJH pore size distribution (inset) of (**a**) CXTi50, (**b**) CXTi70, and (**c**) CXTi90.

Owing to their different morphologies, as indicated by field emission scanning electron microscope (FESEM) images, the samples exhibit porosity in different pore ranges. The porous structure of all samples was studied by N_2_ sorption measurements, and the results are shown in Table 1. The results obtained showed variation in the textural properties of the composites with increasing TiO_2_ content. As previously observed for samples with low TiO_2_ content [11,12], the surface area decreased with the addition of TiO_2_, implying that a proportion of pores in the CX network were blocked by TiO_2_ nanoparticles. Likewise, the surface area of the rest of the samples continued to decrease with the increasing amount of TiO_2_ added, accompanied by a change in the shape of the hysteresis loops, suggesting disordered porosity within the composite structures. This indicates increasingly complex pore systems, due to TiO_2_ aggregates occupying the pore sites, consequently leading to a significant reduction in surface area. According to IUPAC classification, hysteresis loop shapes can be classified, providing insight into the porous networks and adsorption mechanisms [17]. The shape of the isotherm for CXTi50 (Figure 2a) suggests that the porous network comprises wide neck-like or ink bottle-shaped pores, in which pore evaporation is delayed, and desorption at equilibrium does not occur through open pores, while the wide pores remain filled until low p/p_0_ is reached, with evaporation occurring from the neck section, leading to Type H2 hysteresis.

**Figure A2:**
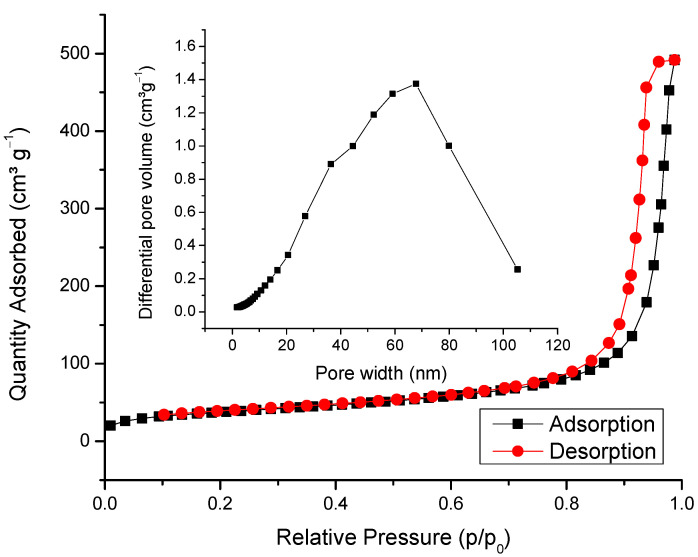
N_2_ isotherm and BJH pore size distribution (inset) of pure TiO_2_.

The nature of the pores can be further classified as H2(a) [18], which means that the neck portion is much narrower than the wider pore cavities, thus generating a sharp drop in the desorption isotherm as the loop closes, indicating pore-blocking effects. Analysis of the shape of the isotherms for CXTi70 (Figure 2b) revealed similar findings. The N_2_ isotherm obtained for the CXTi90 sample (Figure 2c) appears similar to that of the pure TiO_2_, synthesised in this work (Figure A2, Appendix A), where both isotherms are Type H3 with the hysteresis loop confined in the range 0.7 < p/p_0_ < 1.0, demonstrating a wide pore size distribution in the range of 2–100 nm, as also previously reported for low carbon/TiO_2_ composites [19] or pure TiO_2_ nanoparticles [20,21]. This classification of hysteresis implies the existence of aggregates (loose accumulations) of plate-like particles forming slit-like pores [21]. Such characteristics have been reported for mesoporous TiO_2_ nanoparticles synthesised through a sol–gel route for photocatalytic applications [22]. However, the textural properties obtained for TiO_2_ nanoparticles in this study are superior to commercial Degussa P25, exhibiting a specific surface area of ~57 m^2^g^−1^ [23].

**Figure 3:**
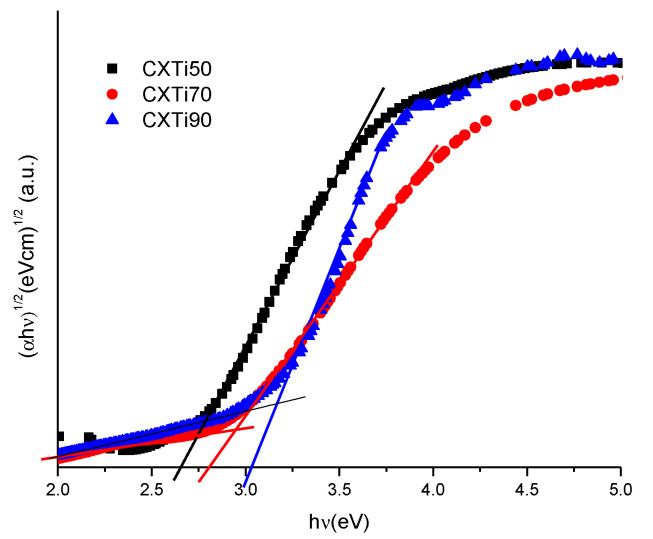
Tauc plots for CXTi composites synthesised within this study.

**Figure 4:**
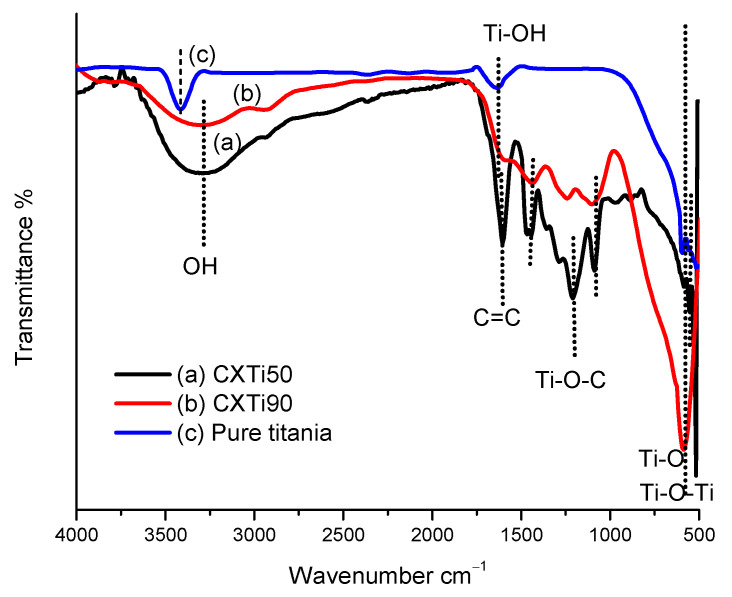
FTIR spectra of (a) CXTi50, (b) CXTi90, and (c) pure titania.

The chemical complexation between CX and TiO_2_ determines the visible light absorption capabilities of the synthesised material for photodegradation under visible light irradiation. The Ti-O-C bond formation introduces a new absorption band in the visible region, whereby the modified electronic structure will require less energy for photoactivation [12,24]. The formation of a charge transfer complex, modification of the electronic structure, charge transfer efficacy, and the consequent optical response are related to the composition of the constituents of the material; hence, the shift of the absorption edge and the lowering of the bandgap are dependent on the amount of CX and TiO_2_ in each sample. The electronic characteristics were studied for newly synthesised composites, and band gaps were calculated using the Tauc method [25]. Figure 3 shows the Tauc plots obtained for the three composites. The calculated band gaps for CXTi50, CXTi70, and CXTi90 were 2.60, 2.93, and 3.10 eV, respectively. As compared to samples with low amounts of TiO_2_ in the composites, the samples synthesised in this work showed increased band gaps, ascribed to the decrease in surface complexation due to the reduced carbon content of these composite samples; hence, the lack of optimal surface complexes between CX and TiO_2_ results in a poor optical response of CXTi samples with very high amounts of TiO_2_. This can also be verified via Fourier-transform infrared spectroscopy (FTIR), and the spectra obtained for two composite materials are shown in Figure 4, with pure TiO_2_ for comparison. Ti-O-C peaks are evident in the spectrum obtained for CXTi50 (trace (a) in Figure 4) in the range from 1200 to 1000 cm^−1^, whereas the spectrum for CXTi90 (trace (b) in Figure 4) shows a diminished peak for chemical bonding between CX and TiO_2_; however, the sample exhibits a prominent Ti-O peak in the fingerprint region, comparable to the spectrum of pure titania shown in trace (c). Other characteristic peaks associated with the functional groups of the synthesised CXTi composites are shown in Table A1 (Appendix A). A consistent correlation between carbonaceous and TiO_2_ contents and their effects on optical response has been previously reported, where the authors asserted that light absorption was reduced with low carbonaceous content in the photocatalyst [26,27]. These studies rationalised the correlation between the mesoporous carbon content and the change in the electronic properties of the composites. Additionally, as observed via FESEM analysis, large amounts of TiO_2_ did not disperse well within these samples, and, therefore, caused the aggregation of TiO_2_ nanoparticles, resulting in increased recombination rates of photogenerated electron/hole pairs, supporting the observation of poor optical response.

### 2.2. Adsorption Performance

**Figure 5:**
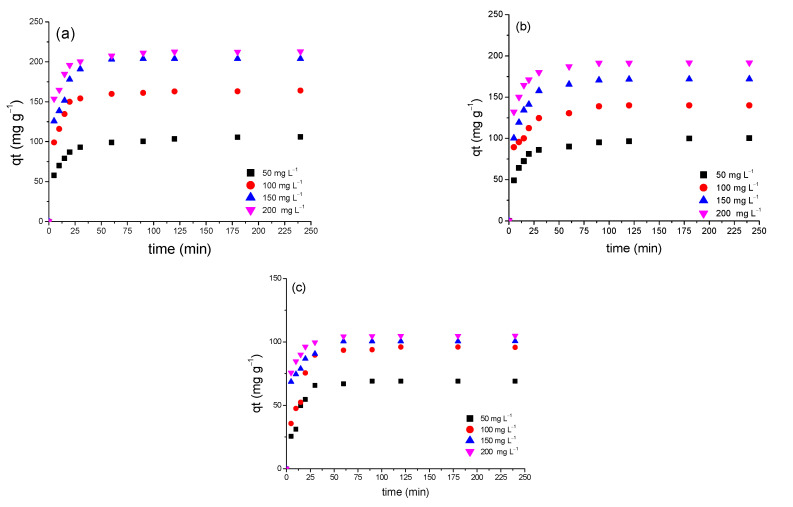
Effect of contact time (0–240 min) to determine adsorption capacity on synthesised composites at initial concentrations of 50, 100, 150, and 200 mg L^−1^ with (**a**) CXTi50, (**b**) CXTi70, and (**c**) CXTi90 (T = 296 K, dose = 0.01 g mL^−1^).

Data for the experimentally determined adsorption capacities of the synthesised samples, as a function of the initial MB concentration (50–200 mg L^−1^) and contact time (0–240 min), were recorded, and are shown in Figure 5a–c. The data obtained show that the trend of adsorption uptake, by all synthesised samples, was similar; that is, the adsorption capacity increased initially, and the process then gradually reached a plateau, as the rate of mass transfer slowed, owing to active sites being saturated, which hindered the adsorption of additional MB molecules on the sample surface; hence, the system attained equilibrium at ~150 min in all cases. Although the adsorption trend is the same for all samples, the extent of adsorption affinity depends predominantly on the nature of the adsorbent, as the change in surface chemistry and porosity play vital roles in adsorption uptake.

The experimentally determined equilibrium adsorption capacities for CXTi50, CXTi70, and CXTi90 are shown in Table 2. Poor uptake with increasing amounts of TiO_2_ in the composites is consistent with the results obtained from surface area and textural analyses, indicating blockage of pores, which results in slower mass diffusion and a reduced number of active sites, resulting in weaker adsorbate–adsorbent interactions. Another detrimental factor for low adsorption on CXTi70 and CXTi90 is the size of the TiO_2_ nanoparticles, or the size of aggregates due to overcrowded TiO_2_ nanoparticles, which may lead to pore blocking, as also observed in other studies [28]. It is noteworthy that the adsorption capacity for low TiO_2_ content analogues (e.g., CXTi10 and CXTi30) were higher than samples synthesised in this work [11,12]. This validates the hypothesis that it is crucial to consider an optimal amount of TiO_2_ deposited in the CX matrix for the pores to be accessible, as well as presence of sufficient surface-active sites for maximum removal performance.

### 2.3. Adsorption Isotherm Analysis

**Figure 6:**
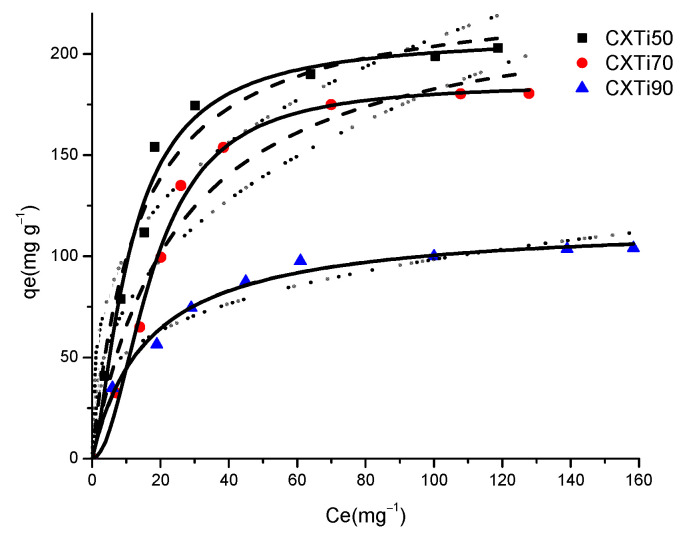
Nonlinear fittings of experimentally obtained adsorption data to Langmuir (dashed line), Freundlich (dotted line), and Sips (solid line) adsorption isotherm models for MB on CXTi50 (■), CXTi70 (●), and CXTi90 (▲) (C_0_ = 100 mg L^−1^, T = 296 K, dose = 0.01 g m L^−1^).

MB adsorption isotherms on CXTi composites are shown in Figure 6. A steep initial increase in MB uptake, with a pronounced slope, was observed for all samples, with an increase in initial concentration of the MB solution (50–200 mg L^−1^). As predicted, the adsorption performance exhibited by each sample was related to the textural properties and surface chemistry of the composites. The surface-active sites originated due to the interaction between CX and TiO_2_, as well as sufficient porosity leading to strong π–π interactions between aromatic groups of the sample and MB molecules. However, due to increasing TiO_2_ loading, the number of surface-active sites is reduced, and blockage of pores results in weakened π–π interactions, and hence, the poor uptake of the MB dye. The maximum adsorption capacity (q_m_) of composites was in the order CXTi50 > CXTi70 > CXTi90.

The experimentally obtained equilibrium adsorption data for MB were analysed using several adsorption isotherm models: Langmuir, Freundlich, and Sips. The isotherm model that demonstrated the most appropriate fit to the experimentally obtained data was selected on the basis of the correlation coefficient (R^2^). The adsorption isotherm models employed in this work were as follows:

The Langmuir isotherm model is a simple theoretical model, which describes monolayer adsorption on homogeneous adsorbents [29]. The model considers several assumptions: (i) there are a well-defined and fixed number of active sites; (ii) adsorption forms a monolayer; (iii) the active sites are identical and cannot host multiple molecules; (iv) the adsorption sites possess the same energy, are energetically equivalent, and therefore, the adsorbent surface is homogenous; (v) the adsorbed molecules do not interact with neighbouring active sites; and (vi) the system is in equilibrium [29,30]. Equation (1) describes the nonlinear Langmuir model:

(1) $$q_{e}=\frac{q_{L}K_{L}C_{e}}{1+C_{e}K_{L}}$$

where q_e_ (mg g^−1^) is the equilibrium adsorbate uptake, C_e_ (mg L^−1^) is the concentration at equilibrium, q_L_ (mg g^−1^) is the quantity of adsorbate corresponding to monolayer coverage, and K_L_ is the Langmuir constant, which indicates the adsorption energy and, consequently, the strength of interactions between the adsorbate and adsorbent.

Furthermore, adsorption favourability can be determined by a dimensionless constant called the separation factor, R_L_, expressed by:

(2) $$R_{L}=\frac{1}{\left(1+K_{L}C_{0}\right)}$$

where C_0_ is the initial adsorptive concentration (mg L^−1^) and K_L_ is the Langmuir constant, which indicates adsorption capacity. R_L_ > 1 suggests that adsorption is unfavourable, while 0 < R_L_ < 1 indicates that adsorption is favourable.

The Freundlich isotherm model can be applied to adsorption processes that occur on highly heterogeneous surfaces. This model assumes that adsorption at multiple sites may occur with multilayer formation, which have a range of adsorption energies, leading to an exponential reduction in energy as surface coverage proceeds. Bond strength is heterogeneous, as a consequence of differences in adsorption site character, or due to already adsorbed molecules. Notably, as a site becomes occupied by an adsorbate molecule, the likelihood of another molecule adsorbing is reduced, since more energy is required. The Freundlich equation can be expressed as:

(3) $$q_{e}=K_{F}C_{e}^{1/\mathrm{nF}}$$

The variables q_e_ (mg g^−1^) and C_e_ (mg L^−1^) are as previously defined for the Langmuir equation. The adsorption constant K_F_ indicates the affinity for adsorption, and n_F_ is related to the scale of the driving force for adsorption, which indicates favourability for adsorption. In summary, 0 < 1/n_F_ < 1 suggests favourable adsorption, 1/n_F_ > 1 indicates unfavourable adsorption, and 1/n_F_ = 1 is obtained for irreversible adsorption. The value of n_F_ also indicates surface/site heterogeneity and provides information about distribution of adsorption energies: 2–10 suggests high adsorption capacity, 1–2 represents moderate adsorption capacity, and a value < 1 suggests low adsorption capacity.

To further understand the adsorption process of MB in the mesopores of RFTi gels, the adsorption data obtained at equilibrium were fitted to an adsorption model based on three parameters. The Langmuir and Freundlich isotherm models have been combined to obtain the Sips isotherm model, which is widely applied, and is represented as:

(4) $$q_{e}=\frac{q_{s}K_{s}C_{e}^{n_{s}}}{1+K_{s}C_{e}^{n_{s}}}$$

The variables q_e_ (mg g^−1^) and C_e_ (mg L^−1^) are as previously defined for the Freundlich and Langmuir equations, 𝐾𝑠 is known as the Sips constant (L g^−1^), and n_s_, the Sips isotherm exponent, indicates the degree of deviation of adsorption from linearity for the adsorption system studied. A value of n_s_ = 1 (or close to) indicates a homogeneous surface for the adsorbent, while n_s_ close to 0 defines a surface with heterogeneously distributed active sites. It is considered an appropriate isotherm model, since it avoids the restriction of increasing concentration, in contrast to the Freundlich isotherm model (which assumes an infinite number of active sites). The Sips isotherm transforms to the Freundlich model at dilute concentrations, while the Sips model reduces to the Langmuir model at higher concentrations, thereby appropriately predicting monolayer adsorption [29,31]. Adsorbent heterogeneity is indicated by 1/n_s_ within the equation; 1/n_s_ < 1 suggests a heterogeneous surface, and 1/n_s_~1 is obtained for homogeneous surfaces [32].

The parameters obtained using the above-described models are given in Table 3. Based on the correlation factor, R^2^, a reasonable fit is obtained for the Langmuir equation, indicating extended monolayer adsorption for the composites, correlated with the textural characteristics of the composites. R_L_ for specific concentrations can be determined using the corresponding values of K_L_, shown in Table 3. According to the values of R_L_ obtained from the application of the Langmuir model, all the systems show favourable adsorption capacity, with values in the range 0 < R_L_ < 1 for all concentrations used within this study. This indicates high and favourable adsorption capacities, as all R_L_ values are low. The values obtained from the Freundlich model, 1/n_F_, are less than one, implying that the dye is favourably adsorbed by the synthesised composites. The value of n_F_ increases with the increase in TiO_2_ in CX, suggesting increasing homogeneity of the TiO_2_ nanoparticles. Overall, it can be observed that the Sips model appropriately predicts the experimentally determined values of adsorption capacity better than the Langmuir and Freundlich models, with higher R^2^ values for all samples. This may be due to the ability of the Sips isotherm model to predict adsorption over wide adsorbate concentration ranges, and also the fact that it accommodates both homogeneous and heterogeneous character in the adsorption system. The values of the heterogeneity factor, n_s_, are greater than one; therefore, the adsorption surface may be predicted to be heterogeneous, with the exception of data obtained for CXTi90. The value of n_s_ determined for CXTi90 is less than one and is characteristic of a homogeneous surface. The Sips model reduces to a Langmuir form when n_s_ = 1; hence, monolayer adsorption for this system can be predicted for this sample [33]. Surface homogeneity of the composite with a very high amount of TiO_2_ in the samples indicates that surface-active sites may be dominated by homogenously distributed functional moieties of TiO_2_.

### 2.4. Photocatalytic Performance

**Figure 7:**
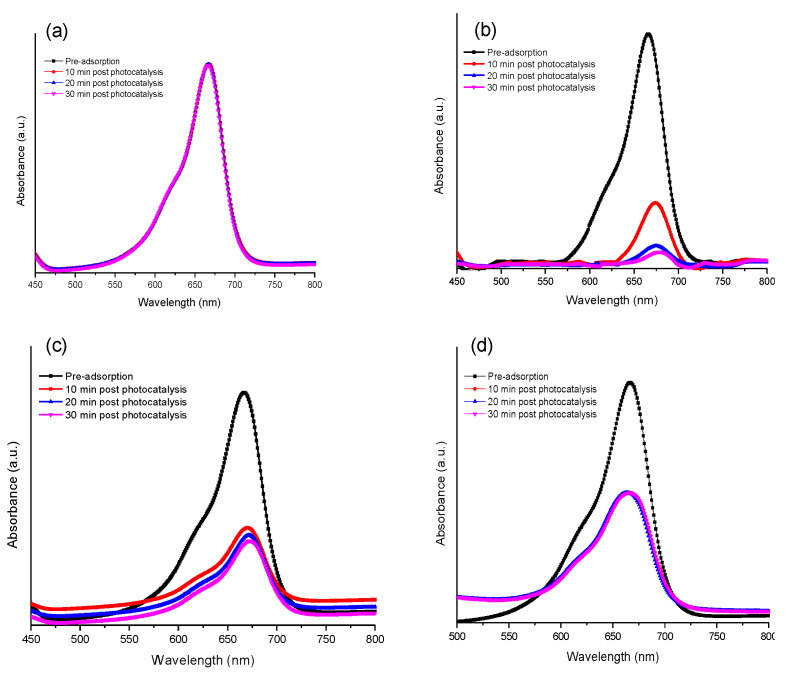
UV-Vis absorption spectra for the degradation of dye in (**a**) the absence of catalyst and by (**b**) CXTi50, (**c**) CXTi70, and (**d**) CXTi90 (experimental conditions: pH~7, temperature 296 K, exposure to visible light after 10 min intervals post adsorption).

**Figure 8:**
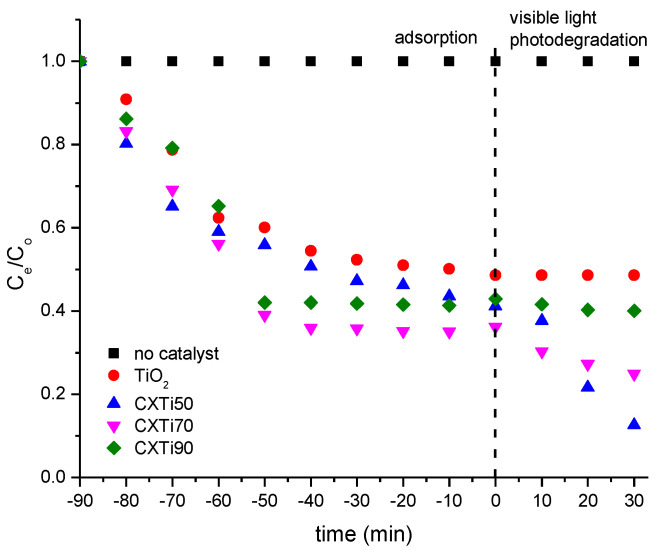
Combined adsorption–photodegradation performance of MB dye degradation tested against synthesised samples (experimental conditions: pH~7, temperature 296 K, exposure to visible light after 120 min).

Figure 7a–d represents MB decolourisation by synthesised composites after exposure to visible light. Post adsorption treatment, this shows a reduction in intensity of the main peak at 663 nm, attributed to the benzene ring and aromatic groups of MB [34]. Upon irradiation with visible light, the absorbance peaks of the MB dye remain almost unchanged in the absence of the catalyst throughout exposure to irradiation (Figure 7a), confirming that MB is stable under visible light [35]. The photodegradation results for synthesised composites are consistent with the adsorption properties and optical responses of the synthesised samples. Additionally, according to the dye degradation curves shown in Figure 7b, CXTi50 showed a significant reduction in absorbance after 30 min of photoactivity, owing to the efficient adsorption–photodegradation exhibited by this composite. However, in the case of CXTi70, the peak reduction was gradual, while no peak reduction was observed for CXTi90, suggesting poor adsorption, a large band gap, and poor optical response exhibited by this composite. The corresponding absorption recorded is plotted in Figure 8 and combined adsorption–photodegradation activity is recorded in Table 4, along with kinetic analysis.

#### Kinetics of Photodegradation

**Figure A3:**
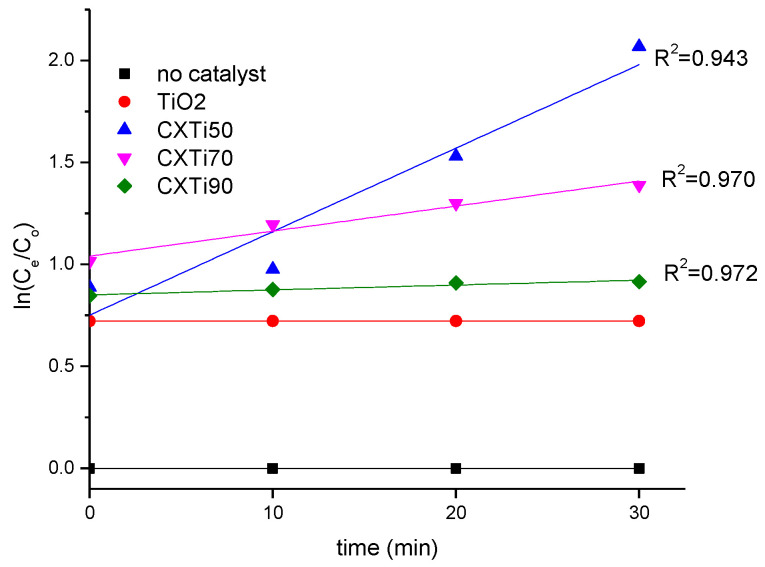
Photocatalytic kinetics of dye degradation for first order linear plot ln (C_e_/C_0_) = f(t).

The decolourisation of MB under visible light was observed by recording dye degradation curves after 10 min post adsorption treatment, as shown in Figure 8. The corresponding absorbance data recorded were fitted to a first order kinetic model:

(5) $$\ln\frac{C_{o}}{C_{e}}=\mathrm{kt}$$

where C_o_ and C_e_ are the MB concentration at zero time and then equilibrated at a given time. Photocatalytic kinetic fits of dye degradation to the first order equation are shown in Figure A3. The value of the rate constant k was evaluated from the gradient of a plot of ln (C_o_/C_e_) vs. time (t) in min. This value correlates with photocatalytic performance, defining the reduction in dye concentration, which is related to the reacting substances, i.e., the photogenerated reactive oxide species; thus, k is higher for greater photocatalytic efficiency.

The synergistic effect of CX and TiO_2_ was analysed by combined adsorption–photodegradation performance, recorded in Table 4. The dye reduction improved from 59 to 87%, 64 to 75%, and 58 to 60% for CXTi50, CXTi70, and CX90, respectively, upon visible light irradiation. Although effective photocatalytic activity is observed for these composites, kinetic analysis showed a decrease in rate constant as TiO_2_ loading increased. Thus, the analysis validates the dependence of TiO_2_ content in the composites and corresponding adsorption–photodegradation responses.

## 3. Conclusions

**Figure 9:**
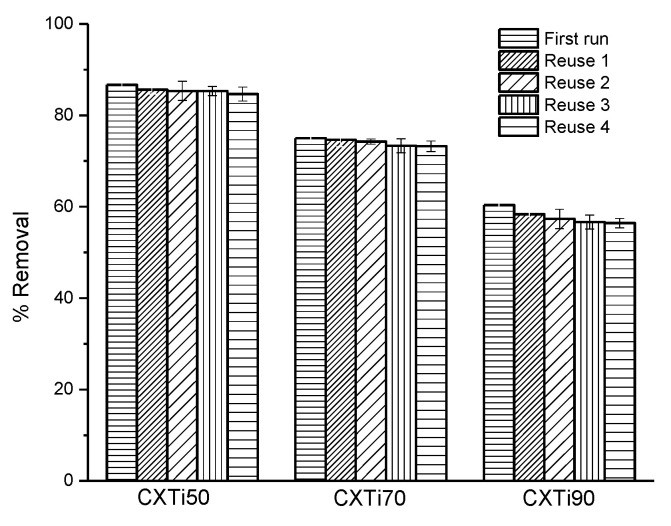
Reusability of synthesised CXTi composites after testing against degradation of MB dye by combined adsorption–photodegradation (C_0_ = 100 mg L^−1^, T = 23 °C, dose = 0.01 g m L^−1^).

A suite of CXTi composites was synthesised using a modified sol–gel technique. The synergy between the carbon xerogel (CX) and TiO_2_ exhibited adsorption–photodegradation activity depending on the amount of TiO_2_ in the composites. The mesoporosity, Ti-O-C complexation, and electronic properties deteriorated due to changing properties, including increasing amounts of TiO_2_ nanoparticles blocking the porous network of CX, insufficient chemical bonding between CX and TiO_2_, and poor response to visible light. Adsorption isotherm analysis showed that the system tended to be homogeneous with a higher loading of TiO_2_ in the composite. All systems were well described by the Sips isotherm model, which indicated that the greatest adsorption capacity was obtained for CXTi50. Composites CXTi50 and CXTi70 were heterogeneous according to the Sips isotherm model, whereas CXTi90 primarily fitted the Langmuir isotherm model equation, suggesting surface homogeneity. Post-adsorption photodegradation was performed under visible light. The results showed improvement from 59 to 87%, 64 to 75%, and 58 to 60% for CXTi50, CXTi70, and CXTi90, respectively. The recyclability of the synthesised composites showed a negligible loss in dye degradation efficiency, indicating a substantial reusability after four repeated cycles (Figure 9). Overall, these composites can efficiently reduce a variety of contaminants owing to their enhanced properties; however, it is essential to balance the amount of TiO_2_ present in terms of site access and performance. Finally, this study provides a framework for the industrial use of these composites in various applications.

## 4. Materials and Methods

### 4.1. Synthesis of Composites

Samples were synthesised following the method described in our previous work [12]. A sol–gel method was used to combine CX with TiO_2_. Table 5 shows the compositions of reagents added to deposit 50, 70, and 90% TiO_2_ in the CX matrix. The reagents used were Resorcinol (R; SigmaAldrich, ReagentPlus, 99%, Poole, UK), formaldehyde (F; 37 wt%), and catalyst Na_2_CO_3_ (C; Sigma-Aldrich, anhydrous, 99.5%, Poole, UK), in the ratios R:F 0.5 and R:C 300. TiO_2_ sol was synthesised using titanium isopropoxide (TTIP) (98+%, ACROS Organics™, Geel, Belgium), in molar ratio 1 TTIP:10 EtOH:0.3 HCl:0.1 H_2_O. A pH~7.4 was maintained using 1M HCl and 1M NaOH. The integrated system was agitated for two hours at 296 K, and then the sol mixture was aged for 72 h at 358 K. After aging, the solvent was exchanged by submerging wet monolithic CXTi in acetone. After 72 h, gels were dried for 48 h at 383 K in a vacuum oven (Townson and Mercer 1425 Digital Vacuum Oven, Stretford, UK), yielding the final CXTi with 50, 70, and 90% TiO_2_.

### 4.2. Structural Characterisation

Thermal gravimetric analysis was performed using a thermal gravimetric analyser (NETZSCH STA 449 F3 Jupiter, Wolverhampton, UK). Al_2_O_3_ crucibles were employed for analysis. A total of ~20 mg of a respective sample was heated to 1073 K at 5 K min^−1^ in N_2_/O_2_ atmosphere. The mass flow controller (MFC) was set to purge gas 1 MFC-50 mL min^−1^ and purge gas 2 MFC-50 mL min^−1^, and protective MFC flow was set to 110% of combined purge gas 1 and 2. The thermographs were obtained using the attached Proteus software for further evaluation, and compositional analysis was carried out according to the ASTM E1131-03 procedure [36]. Morphological analysis was carried out at different magnifications using field emission electron scanning microscopy (FESEM) TESCAN-MIRA (TESCAN'S Essence^TM^ software). Chemical moieties were identified using ABB Fourier-transform infrared (FTIR) spectroscopy (Horizon MB^TM^ FTIR software, MB3000 series, conditions: 400–4000 nm, 4 cm^−1^ intervals, 16 scans). Textural characteristics were studied via N_2_ adsorption at 77 K (Micromeritics ASAP 2420, Hexton, UK) and using the in-built ASAP 2420 software for BET isotherm analysis; BJH theory was used to estimate pore size [37]. Adsorption measurements were obtained using UV-Vis absorption spectra against given wavelengths (Varian Cary 5000 UV-Vis NIR Spectrophotometer, Agilent, UK; Hellma Analytics, Cary WinUV software version 3.0).

### 4.3. Photocatalytic Performance and Adsorption Isotherms

Adsorption behaviour was determined by adding 10 mg of CXTi to 25 mL of prepared MB solutions, with concentrations in the range of 20–200 mg L^−1^. Solution pH was adjusted to ~7, as required, by addition of 1 M HCl and/or 1 M NaOH. Adsorption equilibria were then measured by mixing the solutions and composites, using an orbital shaker (3500 Analog Orbital Shaker unit, 125 rpm, Lutterworth, UK) at 296 K, under dark conditions. Once a predefined period of time had elapsed, the mixture was centrifuged for 15 min, and UV-Vis was conducted on the collected supernatant (Varian Cary 5000 UV-Vis NIR Spectrophotometer, Agilent, UK; Hellma Analytics, Cary WinUV software version 3.0). Similarly, post adsorption, the concentration of dye remaining after photocatalytic treatment was measured using UV-Vis, at predetermined time intervals of irradiation by visible light (irradiance 111 W m^−2^).

The value of q_e_ (mg g^−1^), the equilibrium adsorption capacity, was calculated using:

(6) $$q_{e}=\frac{\left(C_{o}-C_{e}\right).V\left(l\right)}{W}$$

C_o_ and C_e_ are as previously defined. W is adsorbent weight (g), while V is MB solution volume (L).

Contact time can affect adsorption and was investigated by taking aliquots of MB solution in flasks (25 mL, 100 mg L^−1^) and adding 10 mg of composite, before mixing for predetermined contact times (0–240 min). Samples were treated as outlined above for measurement, and adsorption uptake was calculated via Equation (7):

(7) $$q_{t}=\frac{\left(C_{o}-C_{e}\right).V\left(l\right)}{W}$$

C_o_, C_e_, W, and V are as previously defined. Equilibrium concentration was calculated via plots of qt versus time, at which each aliquot was collected, for the range of time intervals used.

## Figures and Tables

**Table 1:** Textural characteristics of the CXTi composites synthesised in this study.

Sample	S_BET_ (m^2^ g^−1^)	Average Pore Size (nm)	Pore Range (nm)	Pore Volume (cm^3^ g^−1^)	% TiO_2_	Ref
RFTi10	439	9	2–57	0.7	11.1	[11]
CXTi30	384	8	2–53	0.8	-	[12]
CXTi50	290	4	2–42	0.2	52	This work
CXTi70	193	5	2–40	0.2	72.5	This work
CXTi90	150	16	2–128	0.4	89	This work

**Table 2:** Experimentally determined equilibrium adsorption capacities of synthesised samples at different initial concentrations (errors omitted as negligible).

	50 mg L^−1^	100 mg L^−1^	150 mg L^−1^	200 mg L^−1^	Ref
RFTi10	109	176	201	212	[11]
CXTi30	113	217	220	221	[12]
CXTi50	100	161	203	211	This work
CXTi70	95	140	171	191	This work
CXTi90	69	95	100	104	This work

**Table 3:** Results of application of the Langmuir, Freundlich, and Sips isotherm models to the adsorption isotherms for MB on CXTi adsorbent gels at 296 K.

Parameters	Sample		
	CXTi50	CXTi70	CXTi90
q_exp_	215	195	104
Langmuir			
q_L_ (mg g^−1^)	231	222	116
K_L_ (Lmg^−1^)	0.108	0.036	0.061
R^2^	0.974	0.958	0.990
Freundlich			
K_F_	47.7	28.1	27.7
n_F_	3.22	2.39	3.60
1/n_F_	0.311	0.420	0.278
R^2^	0.900	0.927	0.951
Sips			
q_s_ (mg g^−1^)	209	185	117
K_s_ (Lmg^−1^)	0.029	0.003	0.064
n_s_	1.45	2.01	0.983
1/n_s_	0.689	0.497	1.017
R^2^	0.983	0.993	0.998

**Table 4:** Summary of combined adsorption–photodegradation performance demonstrated by samples synthesised in this study; data obtained at 296 K.

Sample	Band Gap (eV)	Adsorption (%)	Photodegradation (%)	Rate Constant min^−1^	Ref
RFTi10	2.97	72	75	1.25 × 10^−3^	[11]
CXTi30	2.24	85	99	2.98 × 10^−2^	[12]
CXTi50	2.60	59	87	2.27 × 10^−2^	This work
CXTi70	2.93	64	75	6.95 × 10^−3^	This work
CXTi90	3.10	58	60	3.99 × 10^−4^	This work

**Table 5:** Initial compositions of reagents.

Sample	Resorcinol (g)	Formaldehyde (g)	Catalyst (g)	Titania (g)
CXTi50	3.8756	2.1135	0.0112	6.00
CXTi70	2.3252	1.2681	0.00670	8.40
CXTi90	0.7750	0.4227	0.00224	10.8

## Data Availability

Not applicable.

## Appendix A

**Table A1:** Assignment of additional peaks obtained for FTIR spectra of CXTi50 and CXTi90.

Wavenumber cm^−1^	Assignment
3300	Phenolic OH
1605, 1473	Aromatic ether bridge
1300	C-O-C asymmetric stretching of the methylene ether bridge
1470	CH_2_ (methylene ether bridge)
1200, 1084	Ti-O-C
600	Ti-O-Ti
